# Varied treatment patterns among patients with palmoplantar pustulosis: A claims-based cohort study using data from 2 large US databases

**DOI:** 10.1016/j.jdin.2024.01.007

**Published:** 2024-03-05

**Authors:** Jashin J. Wu, Ran Gao, Rhonda L. Bohn, Anouk Déruaz-Luyet, Stephani Gray, Frank Baehner, Steven R. Feldman

**Affiliations:** a Department of Dermatology, University of Miami, Miller School of Medicine, Miami, Florida; b Boehringer Ingelheim Pharmaceuticals, Inc, Ridgefield, Connecticut; c Bohn Epidemiology, Boston, Massachusetts; d Boehringer Ingelheim International GmbH, Ingelheim, Germany; e Department of Dermatology, Wake Forest University School of Medicine, Winston-Salem, North Carolina

**Keywords:** claims data, database study, palmoplantar pustulosis, treatment patterns

*To the Editor:* Palmoplantar pustulosis (PPP) is a chronic, neutrophilic skin disease characterized by sterile pustules localized to the palms and soles.^1^ Treatment options are largely adopted from those approved to treat plaque psoriasis, despite there being limited evidence on their efficacy for treating PPP.^2^^,^^3^ Due to the rarity of PPP and lack of treatment guidelines,^2^ treatment patterns in the United States are poorly characterized.

This retrospective cohort study used US healthcare claims data from the IBM MarketScan Commercial Database and the Optum Clinformatics Data Mart Database, collected between October 1, 2015, and March 31, 2020 (Supplementary Fig 1, available via Mendeley at https://doi.org/10.17632/db8p7ryc2n.1), to identify PPP treatment patterns. Patients were aged ≥18 years, had newly diagnosed PPP (International Classification of Diseases 10th revision [ICD-10] code L40.3), had ≥1 inpatient or ≥2 outpatient claims 30-180 days apart, and had no PPP claims in the 12 months before diagnosis (Supplementary Methods, available via Mendeley at https://doi.org/10.17632/db8p7ryc2n.1).

Overall, cases of PPP were rare (MarketScan, *n* = 840; Optum *n* = 750) (Supplementary Fig 2, available via Mendeley at https://doi.org/10.17632/db8p7ryc2n.1). The most common comorbidities were plaque psoriasis, diabetes, and obesity (Supplementary Table III, available via Mendeley at https://doi.org/10.17632/db8p7ryc2n.1). Following diagnosis, topical agents were used most often, with topical corticosteroids most frequently used (MarketScan, 36.0%; Optum, 30.3%) (Supplementary Table IV, available via Mendeley at https://doi.org/10.17632/db8p7ryc2n.1). There were 217 (MarketScan) and 228 (Optum) patients who received ≥1 treatment class of interest (biologic, oral systemic steroid, or other oral systemic agent/disease-modifying antirheumatic drugs) with 24 months of follow-up. At Month 1, most patients were receiving treatments that were not classes of interest (MarketScan, 68.7%; Optum, 72.8%). The use of treatment classes of interest increased from Month 1 to Month 24, particularly oral systemic steroids (Supplementary Fig 3, available via Mendeley at https://doi.org/10.17632/db8p7ryc2n.1).

Treatment switching was common in patients from both databases. Use of oral systemic steroid monotherapy increased from Month 3 to Month 24 in both databases (Fig 1). Use of biologic monotherapies steadily increased in MarketScan and fluctuated in Optum during follow-up. Patients commonly switched from monotherapy to combination therapy; at Month 24, combination therapies accounted for 45.0% and 35.7% of treatments, respectively.

**Fig 1:**
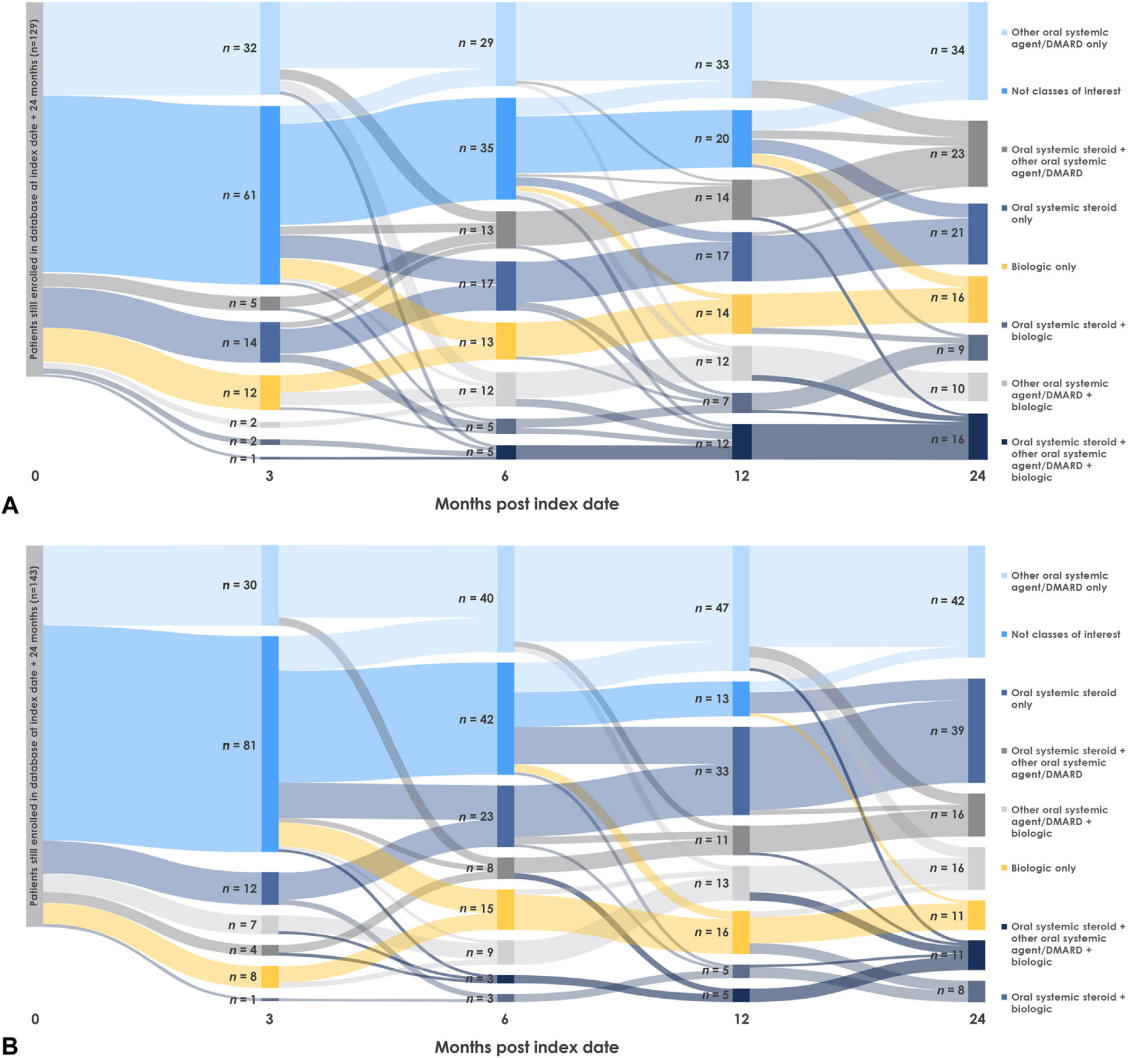
PPP treatment patterns over 24 months in the (**A**) MarketScan and (**B**) Optum databases. No treatment classes of interest were received by 88 patients in MarketScan and 85 patients in Optum over the 24-month follow-up. This figure illustrates the frequency of switching and the lack of any particular sequence of switching in the management of patients with PPP. *DMARD*, Disease-modifying antirheumatic drug, *PPP*, palmoplantar pustulosis.

The lack of established treatment patterns and frequent switching may reflect the shortage of effective, well-tolerated treatments and the paucity of treatment guidelines.^2^ Additionally, frequent switching from monotherapy to combination therapy highlights that some patients may require additive therapies to achieve disease control in the absence of effective monotherapies. However, as the reasons for stopping/switching treatment were not captured in the databases, it is difficult to fully interpret the observed treatment patterns.

The main limitation of this study is that some patients may have been miscoded under the ICD-10 codes for other conditions, including but not limited to general psoriasis (L40), instead of the PPP-specific code (L40.3) and, thus, are missing from this analysis. Additionally, the methodology has not been verified; however, previous studies have reported a similar methodology, including the use of the Optum and MarketScan databases and ICD-10 codes to describe characteristics of patients with psoriasis.^4^

Treatment patterns for patients with newly diagnosed PPP are diverse. With future development of well-tolerated, effective therapies, treatment patterns among patients may become established.

## Conflicts of interest

Jashin J. Wu is or has been an investigator for AbbVie, Amgen, Eli Lilly, Incyte, Janssen, Novartis, and Pfizer; a consultant for AbbVie, Almirall, Amgen, Arcutis, Aristea Therapeutics, Bausch Health, Bayer, Boehringer Ingelheim, Bristol Myers Squibb, Codex Labs, Dermavant, DermTech, Dr Reddy's Laboratories, Eli Lilly, EPI Health, Galderma, Janssen, LEO Pharma, Mindera, Novartis, Regeneron, Samsung Bioepis, Sanofi Genzyme, Solius, Sun Pharmaceutical Industries, UCB, and Zerigo Health, and has been a speaker for AbbVie, Amgen, Bausch Health, EPI Health, Novartis, Regeneron, Sanofi Genzyme, Sun Pharmaceutical Industries, and UCB. Ran Gao is a former employee of Boehringer Ingelheim, and is now an employee of Gilead Sciences. Rhonda L. Bohn is the founder of Bohn Epidemiology, LLC, and has served as a consultant to Boehringer Ingelheim. Anouk Déruaz-Luyet is an employee of Boehringer Ingelheim International GmbH. Stephani Gray declares being a consultant to Boehringer Ingelheim and Bohn Epidemiology, LLC. Frank Baehner is an employee of Boehringer Ingelheim. Steven Feldman declares receiving research, speaking, and/or consulting support from Eli Lilly, GlaxoSmithKline/Stiefel, AbbVie, Janssen, Alvotech, vTv Therapeutics, Bristol Myers Squibb, Samsung, Pfizer, Boehringer Ingelheim, Amgen, Dermavant, Arcutis, Novartis, Novan, UCB, Helsinn, Sun Pharmaceutical Industries, Almirall, Galderma, LEO Pharma, Mylan, Celgene, Valeant, Menlo, Merck, Qurient, Arena Pharmaceuticals, Biocon, Accordant, Argenx, Sanofi, Regeneron, the National Biological Corporation, Caremark, Advance Medical, Suncare Research Laboratories, Informa, UpToDate, and the National Psoriasis Foundation, and is the founder and majority owner of www.DrScore.com [drscore.com] and has stock in Sensal.
